# Cross-cultural adaptation and validation of the Chinese version of the Spine Oncology Study Group Outcomes Questionnaire

**DOI:** 10.3389/fonc.2022.1016258

**Published:** 2022-11-01

**Authors:** Shuheng Zhai, Nanfang Xu, Shanshan Liu, Zhongjun Liu, Xiaoguang Liu, Feng Wei

**Affiliations:** ^1^ Department of Orthopaedics, Peking University Third Hospital, Beijing, China; ^2^ Engineering Research Center of Bone and Joint Precision Medicine, Ministry of Education, Beijing, China; ^3^ Beijing Key Laboratory of Spinal Disease Research, Beijing, China

**Keywords:** spinal metastases, quality of life, questionnaire, reliability, validity

## Abstract

**Background context:**

Patients with spinal metastases always have a poor health-related quality of life (HRQoL) and disease- and treatment-related adverse outcomes. The Spine Oncology Study Group Outcomes Questionnaire (SOSGOQ) has been verified and validated in English for patients with spinal metastases but not in Chinese.

**Purpose:**

This paper aimed to complete the cross-cultural adaptation of the Chinese version of the SOSGOQ, to verify its reliability and validity, and to report on the HRQoL of Chinese patients with spinal metastases.

**Study design/setting:**

This is a single-center, prospective, observational cross-sectional study.

**Patient sample:**

Seventy-six patients were enrolled in this study.

**Outcome measures:**

The SOSGOQ is made up of five HRQoL domains (physical function, neurological function, pain, mental health, social function) and post-therapy questions. The EQ-5D 3L questionnaire covers five items in mobility, self-care, usual activities, pain discomfort, and anxiety-depression, each with three answer options. The SF-36 comprises 36 items divided into eight domains.

**Methods:**

A single-center, prospective, observational cross-sectional study involving patients with spinal metastases who underwent surgery was conducted. HRQoL was evaluated using the Chinese version of the SOSGOQ, the Medical Outcomes Study Questionnaire Short Form 36 Health Survey (SF-36), and the EuroQol 5-Dimension questionnaire (EQ-5D). Demographic, tumor, symptom, and treatment data, as well as Eastern Cooperative Oncology Group (ECOG) information, were collected. Internal consistency reliability, convergent validity, concurrent validity, and clinical validity were used to evaluate reliability. A Spearman’s correlation analysis was used to analyze the relationship between variables.

**Results:**

This study enrolled 76 patients, with a mean age of 55.8 years. The kidney was the most common primary tumor site, and the thoracic spine was the most affected. The internal consistency of the overall SOSQOQ (0.907) was higher than the EQ-5D (0.819), and all items of the SOSQOQ had a high convergent validity (>0.40). The SOSGOQ was significantly correlated with the EQ-5D in respective domains (*p* < 0.001) and overall score (*p* < 0.001), whereas the SF-36 was related to the overall SOSGOQ score and most domains. Total SOSGOG was significantly sensitive to changes in ECOG (*p* = 0.017), prior surgery (*p* = 0.001), and tumor type (*p* = 0.026).

**Conclusions:**

The SOSGOQ is a reliable and effective tool for evaluating HRQoL in patients with spinal metastases, with high sensitivity and specificity. Surgical treatment can significantly improve patients’ HRQoL.

## Background

Patients with cancer experience poor health-related quality of life (HRQoL) with disease- and treatment-related adverse outcomes (1). Spinal metastasis has a specific and significant impact on HRQoL, especially in the neural function and pain domains (2). Neurological dysfunction caused by compression includes the inability to ambulate, dysfunction of the bowel and bladder systems, or both (3). Pain is caused by mechanical instability due to spinal metastases invading the vertebral body (4).

HRQoL was a critical component in assessing the efficacy of cancer therapies, which was recognized by international cancer research organizations (5, 6). Surgery as a common type of cancer therapy is necessary for spinal metastasis when it comes to indicated-neural compression, pathological fracture, instability, and progressive deformity, and it cannot be replaced by other treatments (7). The question of whether surgery can improve or maintain HRQoL is important because improving HRQoL for the rest of a patient’s life is the main treatment goal for patients with spinal metastases (8, 9).

There are many general and specific questionnaires used to assess HRQoL in patients with metastatic spine disease (10). The Medical Outcomes Study Questionnaire Short Form 36 Health Survey (SF-36) and the EuroQol 5-Dimension questionnaire (EQ-5D) are recommended as general questionnaires for disease comparison (10, 11). As the first spine oncology-specific HRQoL measure, the Spine Oncology Study Group Outcomes Questionnaire (SOSGOQ) was verified to be sensitive and specific for detecting changes in patients with spine tumors (12). Moreover, the SOSGOQ has been evaluated by AOSpine Knowledge Forum Tumor in an international, multicenter, prospective observational cohort study (9).

The SOSGOQ was developed and validated in English and has not been translated into other languages. The aim of the study was to carry on the process of cultural adaptation of the SOSGOQ into Chinese and to evaluate its validity in patients with spinal metastasis.

## Method

### Translation

For translation and cross-cultural adaptation, we followed the widely accepted guidelines set up by the International Quality of Life Assessment (IQOLA) project (13). Independent forward translation, review, reconciliation of differences, reverse translation, and establishment of the consensus-based prefinal version by an expert panel have been done.

### Design

A single-center, prospective, observational cross-sectional study was conducted from March to December 2019 in the orthopedic department of our outpatient clinic. Patients were eligible for inclusion if they had been diagnosed or suspected of having spinal metastasis. The research assistants sent the Chinese versions of the SOSGOQ, EQ-5D, and SF-36 to the patients to ensure that the questionnaires were filled out completely (11). After, the questionnaires were collected and entered into a specific database. Other information about patients was obtained from the medical report system and included their age at the time of survey completion, gender, primary tumor type based on the pathological report, affected anatomical location, presence of other bone metastases and visceral metastases, presence of pain, symptoms of neurological compromise, Eastern Cooperative Oncology Group (ECOG) performance status, prior surgery, and radiotherapy. Patients without a pathological report or whose pathological report did not indicate metastasis were excluded. The pathological report was obtained as a result of a subsequent surgery or biopsy. Patients who did not provide complete information were also excluded. The study included 76 patients, all of whom provided informed consent.

### Outcome measures

The SOSGOQ is made up of five HRQoL domains (physical function, neurological function, pain, mental health, social function) and post-therapy questions. Individual SOSGOQ items were scored on a scale of 0 (best) to 4 (worst). The overall score of the SOSGOQ was calculated by adding the points from all items in the five HRQoL domains (10).

The EQ-5D 3L questionnaire covers five items in mobility, self-care, usual activities, pain discomfort, and anxiety-depression, each with three answer options. An overall EQ-5D index was calculated for those who completed all five questions using an algorithm described by Gordon et al. (11).

The SF-36 comprises 36 items in eight domains: physical functioning, role-physical, bodily pain, general health, vitality, social functioning, role-emotional, and mental health. All domain scores were transformed to a scale of 0 to 100, in which a higher score represents a better QOL.

### Statistical analysis

The baseline characteristics were summarized using descriptive statistics. Internal consistency was assessed using the Cronbach’s alpha coefficient, which ranges from 0 to 1, with a higher score indicating greater internal consistency. A Cronbach’s alpha above 0.70 is recommended; however, a high Cronbach’s alpha (above 0.90) may indicate question redundancy (14–16). Convergent validity was evaluated by correlating the item score with the total score of its own domain, whereas divergent validity was evaluated by correlating the item score to the total score of the other SOSGOQ domains (Spearman’s rank). A scaling success was defined as an item correlation with its own domain that was significantly higher than the other SOSGOQ domains, with a minimum correlation of 0.40 (17). The concurrent validity of the SOSGOQ domains was evaluated using Spearman’s rank correlation with the domains of the SF-36 and the EQ-5D score. The domains that are conceptually related were expected to demonstrate a correlation of at least 0.40. Clinical validity was examined using the ability of the SOSGOQ to discriminate between patient groups. Patients with an ECOG performance score of 0 or 1 were compared to those with an ECOG score of 2. Patients with prior surgery were compared to those who had not. SPSS 26.0 software was used for statistical analysis, with a significance level of *p* < 0.05.

## Results

From March to December 2019, 76 consecutive Chinese-speaking patients with metastatic spine disease (28 women and 48 men) with a mean age of 55.8 years (standard deviation (SD), 11.3) were enrolled. The kidney (23.7%) was the most common primary tumor site, followed by the lung (19.7%). The thoracic spine was the most commonly affected (48.7%). Most patients had pain (78.9%), 39 patients (51.3%) had neurological compromise, and there was substantial variability in ECOG performance status. Of the 76 patients, 39 underwent surgery with or without additional radiotherapy, while the remaining patients had not yet received therapy. There were no patients who only received radiotherapy (**Table 1**).

**Table 1:** Summary of baseline characteristics.

Characteristics (*n* = 76)	No. (%)
Age at treatment (year)^a^	55.8 (11.3)
Sex	
Female	28 (36.8)
Male	48 (63.2)
Primary tumor	
Kidney	18 (23.7)
Lung	15 (19.7)
Breast	11 (14.5)
Thyroid	10 (13.2)
Other	22 (29.0)
Location	
Cervical	28 (36.8)
Thoracic	37 (48.7)
Lumbar	17 (22.4)
ECOG PS	
0–1	48 (63.2)
2–4	28 (36.8)
Pain	60 (78.9)
Symptoms of neurological compromise	39 (51.3)
Prior surgery for metastatic spine disease	41 (54.0)

**Table 2:** Item completion rate, internal consistency, floor and ceiling effect, and score distribution per domain of the SOSGOQ and for the EQ-5D 3L.

Overall	Number of items per scale	Median (interquartile range)	Range	Cronbach alpha	Floor effect *n* (%)	Ceiling effect *n* (%)
SOSGOQ	20	36 (24)	7–66	0.907	0 (0)	0 (0)
Domains within SOSGOQ						
Physical function	4	8 (8)	0–16	0.916	5 (6.7)	5 (6.7)
Neurological function	4	4 (5)	0–16	0.727	8 (10.7)	2 (2.7)
Pain	4	9 (5)	0–16	0.765	1 (1.3)	1 (1.3)
Mental health	4	7 (4)	1–13	0.578	0 (0)	0 (0)
Social function	4	8 (5)	1–15	0.496	0 (0)	0 (0)
Post-therapy questions (*n* = 41)	7	7 (5.5)	1–25	0.837	0 (0)	0 (0)
EQ-5D 3L score	5	0.57 (0.46)	−0.15–0.94	0.819	1 (1.3)	10 (13.2)

There were seven items (0.4%) missing. In the SOSQOQ, items in the neurological function domain (items 5, 6, 7, and 8) and the item addressing depressed feeling (item 13) demonstrated positive skewness, with 22%, 12%, 28%, 16%, and 8% of the patients reporting moderate to severe symptoms, respectively. In the EQ-5D 3L questionnaire, the items addressing mobility, usual activities, pain discomfort, and anxiety-depression (items 1, 3, 4, and 5) demonstrated positive skewness.

There were no floor effects or ceiling effects observed in the SOSGOQ total score, whereas the EQ-5D had a floor effect of 1.3% and a ceiling effect of 13.2%. The physical function, neurological function, and pain domains in the SOSGOQ all had floor effects and ceiling effects, with the neurological function domain having the largest (floor effect, 10.7%).

The internal consistency of the overall SOSQOQ (0.907) was higher as compared to the internal consistency of the EQ-5D (0.819). Internal consistency as determined by Cronbach’s α per domain ranged from 0.496 (social function) to 0.916 (physical function).

Convergent validity was above the threshold of 0.40 for all items. All items were found to have a stronger correlation with their own domain compared to the other domains. Items 12 (How confident do you feel about your ability to manage your pain on your own)?, 15 (Do you have a lot of energy)?, 19 (Are you comfortable meeting new people)?, and 20 (Do you leave the house for social functions)? did not have satisfactory (*r* < 0.40) item-total correlations (**Table 3**).

**Table 3:** Multitrait analysis: assessing convergent and divergent validity of individual items with the general five domains of the Spine Oncology Study Group Outcomes Questionnaire (SOSGOQ) and the overall SOSGOQ.

Domains	Item number	Physical function	Neurological function	Pain	Mental health	Social function	Overall SOSGOQ score
Physical function	Item 1	**0.896**	0.642	0.520	0.384	0.424	0.768
	Item 2	**0.900**	0.644	0.630	0.434	0.550	0.822
	Item 3	**0.877**	0.652	0.533	0.466	0.393	0.778
	Item 4	**0.907**	0.677	0.607	0.443	0.480	0.814
Neurological function	Item 5	0.405	**0.720**	0.225	0.262	0.082	0.446
	Item 6	0.337	**0.521**	0.306	0.388	0.258	0.453
	Item 7	0.767	**0.821**	0.522	0.415	0.349	0.762
	Item 8	0.430	**0.687**	0.389	0.346	0.311	0.556
Pain	Item 9	0.604	0.438	**0.844**	0.496	0.380	0.689
	Item 10	0.513	0.512	**0.802**	0.543	0.338	0.659
	Item 11	0.771	0.638	**0.783**	0.538	0.512	0.825
	Item 12	0.116	0.133	**0.611**	0.589	0.221	0.369
Mental health	Item 13	0.174	0.380	0.306	**0.636**	0.219	0.408
	Item 14	0.387	0.295	0.526	**0.717**	0.233	0.514
	Item 15	0.202	0.191	0.266	**0.467**	0.296	0.322
	Item 16	0.476	0.442	0.727	**0.815**	0.493	0.707
Social function	Item 17	0.574	0.557	0.569	0.618	**0.724**	0.739
	Item 18	0.532	0.374	0.435	0.504	**0.673**	0.630
	Item 19	0.120	0.077	0.010	-0.041	**0.521**	0.172
	Item 20	0.125	−0.007	0.114	0.038	**0.640**	0.205

Bold values mean convergent validity, indicated the correlation between all items and their own domain.

In terms of concurrent validity, the SOSGOQ was significantly correlated with EQ-5D in both respective domains (*p* < 0.001) and overall score (*p* < 0.001). Moreover, the respective domains of SF-36 were highly related to the overall SOSGOQ score, as were the majority of the SOSGOQ domains (**Table 4**).

**Table 4:** Concurrent validity with the SF-36 and EQ-5D.

Reference score	SOSGOQ physical function	SOSGOQ neurological function	SOSGOQ pain	SOSGOQ mental health	SOSGOQ social function	SOSGOQ
SF-36						
Physical functioning	−0.812 (<0.001)	−0.647 (<0.001)	−0.537 (<0.001)	−0.592 (<0.001)	−0.603 (<0.001)	−0.774 (<0.001)
Role—physical	−0.507 (<0.001)	−0.256 (0.121)	−0.311 (0.058)	−0.214 (0.198)	−0.358 (0.027)	−0.407 (0.011)
Bodily pain	−0.854 (<0.001)	−0.686 (<0.001)	−0.668 (<0.001)	−0.640 (<0.001)	−0.572 (<0.001)	−0.809 (<0.001)
General health	−0.479 (0.002)	−0.461 (0.004)	−0.428 (0.007)	−0.519 (0.001)	−0.245 (0.138)	−0.488 (0.002)
Vitality	−0.604 (<0.001)	−0.474 (0.003)	−0.517 (0.001)	−0.619 (<0.001)	−0.526 (0.001)	−0.636 (<0.001)
Social functioning	−0.783 (<0.001)	−0.499 (0.001)	−0.440 (0.006)	−0.385 (0.017)	−0.490 (0.002)	−0.618 (<0.001)
Role—emotional	−0.468 (0.003)	−0.330 (0.043)	−0.301 (0.066)	−0.138 (0.407)	−0.348 (0.032)	−0.412 (0.010)
Mental health	−0.557 (<0.001)	−0.537 (0.001)	−0.552 (<0.001)	−0.667 (<0.001)	−0.707 (<0.001)	−0.694 (<0.001)
EQ-5D 3L score	−0.735 (<0.001)	−0.626 (<0.001)	−0.593 (<0.001)	−0.480 (<0.001)	−0.437 (<0.001)	−0.753 (<0.001)

In **Table 5**, total SOSGOG was significantly sensitive to changes in domains of ECOG (*p* = 0.017), prior surgery (*p* = 0.001), and tumor type (*p* = 0.026). For the respective domains, physical function and neurological function were correlated with a change in ECOG score. Furthermore, prior surgery was significantly linked to physical function, pain, and mental health, whereas tumor type was linked to physical function, neurological function, and pain. In addition, EQ-5D was also sensitive to changes in ECOG (*p* = 0.020) and prior surgery (*p* = 0.001), but not in tumor type (*p* = 0.098).

**Table 5:** Sensitivity to changes in the SOSGOQ based on changes in ECOG score.

Change in domain	Physical function	Neurological function	Pain	Mental health	Social function	Total SOSGOQ	EQ-5D
ECOG							
ECOG good (0–1)	8.0 (5.6)	3.2 (3.2)	10.6 (2.6)	7.9 (2.5)	7.0 (3.5)	36.7 (14.0)	0.554 (0.267)
ECOG poor (2–4)	11.8 (3.6)	8.1 (3.6)	10.4 (3.3)	7.8 (3.0)	8.7 (3.1)	46.7 (10.8)	0.350 (0.255)
*p*	0.016	<0.001	0.808	0.900	0.101	0.017	0.020
Prior surgery							
Yes	6.6 (4.6)	3.9 (3.5)	7.3 (3.6)	6.2 (3.0)	7.1 (2.9)	31.1 (14.6)	0.671 (0.267)
No	9.9 (5.0)	5.4 (4.2)	10.6 (2.7)	7.9 (2.7)	8.1 (3.4)	42.0 (13.0)	0.449 (0.268)
*p*	0.004	0.087	<0.001	0.015	0.152	0.001	0.001
Tumor type							
Slow growth	5.6 (4.5)	2.8 (2.9)	7.3 (3.2)	6.3 (2.9)	6.8 (3.7)	28.8 (14.0)	0.694 (0.251)
Moderate growth	9.8 (4.6)	5.9 (4.4)	9.2 (3.8)	6.8 (2.9)	7.6 (2.9)	39.3 (14.3)	0.537 (0.273)
Rapid growth	8.4 (5.2)	4.4 (3.4)	8.8 (3.6)	7.5 (3.0)	7.5 (3.2)	35.2 (14.7)	0.556 (0.269)
*p*	0.019	0.020	0.030	0.347	0.373	0.026	0.098

## Discussion

With the effective control of the primary tumor and the improvement of adjuvant therapy (radiotherapy, chemotherapy, and targeted therapy), patients with spinal metastases usually have a longer overall survival time, and the evaluation of HRQoL is of particular concern. Therefore, more and more studies have been done on the HRQoL of spinal metastases. Yao et al. reviewed 24 articles and found that surgery can greatly improve the HRQoL of patients with spinal metastases, as a first-line treatment for restoring neurological function and relieving pain (18). In a cohort of 239 patients with spinal metastases, Barzilai et al. proposed that surgery can stabilize or improve neurological function, thereby improving HRQoL (19). This study has completed the Chinese version of the SOSGOQ according to the process of cross-cultural adaptation and verified its internal consistency reliability, convergent validity, concurrent validity, and clinical validity. We also compared the specific SOSGOQ with the general questionnaires (SF-36 and EQ-5D) and reported HRQoL in Chinese patients with spinal metastatic disease.

We found a significant correlation between the specific SOSGOQ and general questionnaires, indicating good HRQoL performance. In our study, satisfactory internal consistency reliability, convergent validity, concurrent validity, and clinical validity were shown with SOSGOQ. The Cronbach’s α coefficients indicated a higher internal consistency of the SOSGOQ than EQ-5D (**Table 2**). Although the internal consistency of the SOSGOQ (0.907) was above the 0.90 threshold, inter-item correlation analysis within the SOSGOQ refuted possible item redundancy as all correlations were below the 0.80 thresholds (**Table 3**). All items had good convergent validity by comparing their own domain with other domains. Moreover, all but four items (12, 15, 19, 20) had high correlations with the overall SOSGOQ score. Compared with SF-36 and EQ-5D, SOSGOQ revealed high concurrent validity and consistency (**Table 4**). Items 12 (How confident do you feel about your ability to manage your pain on your own)? and 15 (Do you have a lot of energy)? may be too sharp and subjective for patients, whose answers almost tended to be “No.” Items 19 (Are you comfortable meeting new people)? and 20 (Do you leave the house for social functions)? may lack specificity and be inappropriate for patients, especially for those with spinal metastases. The same with EQ-5D, the SOSGOQ showed significant sensitivity to patients with different ECOG scores, implying high clinical validity (**Table 5**). However, apart from the total SOSGOQ score, only the domains of physical function and neurological function were associated with ECOG scores. This might be because ECOG criteria depended on physical and neurological conditions, while pain, mental state, and social ability were not. Patients with poor neurological function may also have a good mental state and less pain. Referring to tumor types, only SOSGOQ had a significant correlation rather than EQ-5D, which explained that EQ-5D had poor specificity for spinal metastases. There were no floor effect and ceiling effects observed in the SOSGOQ total score, while the EQ-5D had a floor effect of 1.3% and a ceiling effect of 13.2%, which indicated patients with spinal metastases were well measured with SOSGOQ rather than EQ-5D (**Table 2**). As a result, the SOSGOQ had better reliability and validity to assess HRQoL in patients with spinal metastases. In addition to the high sensitivity, SOSGOQ showed significant specificity, while EQ-5D and SF-36 did not.

For reported HRQoL in Chinese patients with spinal metastases, the surgical treatment appeared to have a better SOSGOQ score than the nonsurgical treatment. Michael et al. analyzed a study of 142 patients with spinal metastases undergoing surgical treatment and found that HRQoL improved at 6 weeks, 3 months, 6 months, and 12 months after surgery (20). Miyazaki et al. suggested that compared with the nonsurgical group, both HRQoL and prognosis were improved in the surgical group (21). In Leonard’s study, 92 patients with spinal metastases treated surgically had a significant long-term improvement in HRQoL (22). By improving physical function and mental health and relieving pain, surgery could significantly improve the HRQoL (23). In addition, the type of primary tumor could also affect HRQoL. Rapid growth of spinal metastases usually resulted in more severe physical dysfunction, neurological dysfunction, and pain, which predicts a poor prognosis and low HRQoL (18, 24).

## Conclusion

In conclusion, the Chinese version of SOSGOQ has been proven to have high reliability and validity. Compared to EQ-5D and SF-36, SOSGOQ had significantly higher sensitivity and specificity for patients with spinal metastases. It will be recommended for application in Chinese-speaking patients with spinal metastases.

There were also a few limitations to this study. Firstly, this was a single-center study. TO investigate, multicenter studies should be performed. Secondly, the patients included in our study were all outpatients, which might result in selection bias. It reflected the patients’ surgeons faced, instead of all patients with spinal metastases. Thirdly, because of the low incidence of spinal metastases, we included a small sample size, and it was difficult to enroll a large number. Fourthly, limited to the course of treatment, we did not evaluate test–retest reliability.

## Data availability statement

The datasets generated and/or analyzed during the current study are not publicly available but are available from the corresponding author on reasonable request. Requests to access these datasets should be directed to weifeng@bjmu.edu.cn.

## Ethics statement

Written informed consent was obtained from the individual(s) for the publication of any potentially identifiable images or data included in this article.

## Author contributions

FW and XL contributed to the study’s conception and design. Material preparation, data collection, and analysis were performed by SZ, NX, and SL. The first draft of the manuscript was written by SZ and SL. FW, ZL, and XL revised the manuscript. All authors contributed to the article and approved the submitted version.

## Funding

This study was funded by the National Natural Science Foundation of China (Code: 82172395). This funding source had no role in the design of this study and will not have any role during its execution, analyses, interpretation of the data, or decision to submit results. External peer review took place during the funding process.

## Conflict of interest

The authors declare that the research was conducted in the absence of any commercial or financial relationships that could be construed as a potential conflict of interest.

## Publisher’s note

All claims expressed in this article are solely those of the authors and do not necessarily represent those of their affiliated organizations, or those of the publisher, the editors and the reviewers. Any product that may be evaluated in this article, or claim that may be made by its manufacturer, is not guaranteed or endorsed by the publisher.

## Abbreviations

HRQoL, health-related quality of life; SF-36, Medical Outcomes Study Questionnaire Short Form 36 Health Survey; EQ-5D, EuroQol 5 Dimensions questionnaire; SOSGOQ, The Spine Oncology Study Group Outcomes Questionnaire; IQOLA, International Quality of Life Assessment; ECOG, Eastern Cooperative Oncology Group; SD, standard deviation.
